# Tissue-Derived Stem Cell Research 2019

**DOI:** 10.1155/2019/4765684

**Published:** 2019-12-19

**Authors:** Ming Li, Kequan Guo, Luca Vanella

**Affiliations:** ^1^Laboratory of Cardiovascular Disease, Novel, Non-Invasive, and Nutritional Therapeutics, Graduate School of Medicine, Osaka University, Osaka 5650874, Japan; ^2^Department of Cardiac Surgery, Beijing Institute of Heart, Lung & Blood Vessel Disease, Beijing Anzhen Hospital, Capital Medical University, Beijing 100029, China; ^3^Department of Drug Science, Section of Biochemistry, University of Catania, 95125 Catania, Italy

Tissue-derived stem cells (TDSCs) have proven to be a feasible source of cells for tissue regeneration medicine in recent experimental and clinical studies. TDSCs are presented in tissues such as bone marrow (BM), blood vessels, adipose tissues, and placental and dental pulp for repairing damaged areas by generation of new cells and tissues. Mesenchymal stem cells (MSCs) are multipotent stem cells, reported to present plastic adherence and different abilities in endodermal, mesodermal, and ectodermal cells and immunoregulatory property potential in cell-based therapies and regenerative medicine. To better understand TDSCs, we opened up the special issue focused on TDSCs in 2016 and pushed forward an annual issue in 2019 including recent findings concerning TDSCs.

In the special issue, 16 selected papers including 13 research papers and 3 review papers were published. Nine groups reported their studies in MSCs derived from BM, adipose, placental, and valve. M. Alimandi et al. reported that clonogenicity and human BM-MSC expansion are two distinct biological events and quorum sensing may operate in BM-MSC cultures and determine the potential growth of clonal strains. H. Fabre et al. discussed the first comparison of detailed immunophenotypic analysis and chondrogenic differentiation potential of human BM, Wharton's jelly, dental pulp, and adipose tissue MSCs performed under the same serum-free conditions. X. Wang et al. indicated that fetal dermal MSC exosomes may promote wound healing by activating the adult dermal fibroblast cell motility and secretion ability via the Notch signaling pathway. M. B. Avery et al. reported that allogenic BM-MSCs had the ability to prevent aneurysm formation in a known rabbit elastase aneurysm model. W. Lu et al. reported that the mesenchymal characteristics of skeletal progenitor cells within different regions of long bones showed some differences. Periosteal mesenchymal progenitors showed a higher proliferative ability and adipogenic differentiation potential. In contrast, endosteal mesenchymal progenitors were more prone to osteogenic differentiation. Y. Huang et al. reported that novel rapidly proliferating valve-derived stroma cells with fibroblast morphology, which were found to express mesenchymal and osteogenic markers, may contribute to aortic valve calcification. P. Monsarrat et al.'s research showed antibacterial effects of human adipose-derived stroma cells by phagocytosis and secretion of oxygenated free radicals and antibacterial molecules. D. Aboalola et al. indicated the complex interactions between IGFBP-6 and IGFs in placental MSC differentiation into the skeletal muscle and that the IGF signaling axis, specifically involving IGFBP-6, is important in muscle differentiation. M. E. Castro-Manrreza et al. discussed presence and biological characteristics of MSCs in the epidermis and dermis of psoriasis patients.

Three groups reported their researches related to human regulatory macrophage, Kupffer cells, and bulk composite resins. L. Hummitzsch et al. reported that human regulatory macrophages (Mreg) may prove to be beneficial as a cell therapy-based treatment option for ischemia/reperfusion-associated illnesses. However, donor characteristics seem to crucially influence the effectiveness of Mreg treatment. D. Meng et al. discussed a critical role of Kupffer cells in the maintenance and promotion of adult mouse liver hematopoiesis. S.-M. Lee et al. discussed a difference in depth of cytotoxicity and antidifferentiation between the bulkfill composite resins, which was mainly due to different cure depths and ingredients. P. N. Rao et al. reported that deceased donor-derived stem cells may be a viable alternative to living donor stem cells.

S. Liu et al. summarized the characteristic of spinal cord endogenous neural stem cells, especially their properties after injury. L. Nevi et al. reviewed the new strategies that have been adopted to improve cell grafting and track cells after transplantation. F. Sallustio et al. analyzed many different functions that the Toll-like receptors (TLRs) assume in stem/progenitor cells, pointing out that they can have different effects, depending on the background and the kind of ligands that they recognize. They also discussed the TLR involvement in the response of stem cell to specific tissue damage.

We hope that the annual special issue provided insights into TDSCs such as MSCs and speeds up their clinical application in regenerative trials in the near future.

